# Supplementary material for: Blood Pressure Estimation Using On-body Continuous Wave Radar and Photoplethysmogram in Various Posture and Exercise Conditions
Source: Sci Rep. 2019 Nov 8;9:16346. doi: 10.1038/s41598-019-52710-8 (PMC6841972; doi:10.1038/s41598-019-52710-8)
Supplement: Supplementary file 2 — Article.pdf file [file 41598_2019_52710_MOESM2_ESM.pdf]

Blood Pressure Estimation Using On-body Continuous Wave Radar and Photoplethysmogram in Various Posture and Exercise Conditions

Malikeh Pour Ebrahim[1], Fatemeh Heydari[1], Taiyang Wu[1], Katherine Walker[2], Keith Joe[2], Jean-Michel Redoute[1], and Mehmet Rasit Yuce[1,*]

[1]Department of Electrical and Computer Systems Engineering, Monash University, Melbourne, Australia
[2]Emergency Department, Cabrini Health, Melbourne, Australia
[*]mehmet.yuce@monash.edu

ABSTRACT

The pulse arrival time (PAT), pre-ejection period (PEP) and pulse transit time (PTT) are calculated using on-body continuous wave radar (CWR), Photoplethysmogram (PPG) and Electrocardiogram (ECG) sensors for wearable continuous systolic blood pressure (SBP) measurements. The CWR and PPG sensors are placed on the sternum and left earlobe respectively. This paper presents a signal processing method based on wavelet transform and adaptive filtering to remove noise from CWR signals. Experimental data are collected from 43 subjects in various static postures and 26 subjects doing 6 different exercise tasks. Two mathematical models are used to calculate SBPs from PTTs/PATs. For 38 subjects participating in posture tasks, the best cumulative error percentage (CEP) is 92.28% and for 21 subjects participating in exercise tasks, the best CEP is 82.61%. The results show the proposed method is promising in estimating SBP using PTT. Additionally, removing PEP from PAT leads to improving results by around 9%. The CWR sensors present a low-power, continuous and potentially wearable system with minimal body contact to monitor aortic valve mechanical activities directly. Results of this study, of wearable radar sensors, demonstrate the potential superiority of CWR-based PEP extraction for various medical monitoring applications, including BP measurement.

Introduction

Blood Pressure (BP) abnormalities, such as hypotension and hypertension, are important risk factors for many short- and long-term critical illnesses, with a global disease burden of about 1.25 billion people[1]. The gold standard for BP measurement is the use of a cuffed sphygmomanometer[2]. However, cuff measurements are uncomfortable and create barriers to measuring BPs[3] over time. In addition, BP measurement with sphygmomanometry can be inaccurate[4]. Sometimes continuous BP measurement is required. In ambulatory, community and domestic settings, continuous BP measurement improves accuracy in diagnosis of chronic hypertension[5]. When continuous BP measurement is required in critically ill patients, more invasive methods of BP monitoring are used (arterial lines), which can cause infection and loss of limbs due to ischaemia[6].

Several BP monitoring devices have been proposed, based on Pulse Arrival Time (PAT), Pulse Transit Time (PTT) and Pulse Wave Velocity (PWV). These all use different devices and data analysis methods. A photoplethysmograph (PPG) is one of the most popular sensors[7]. Ballistocardiography (BCG) and PPG[8], mobile phone sensors[9], microelectromechanical sensors[10], magneto-plethysmographic sensors[11], bio watches (a wrist-based BP monitor)[12], wearable glasses[13] and bio-impedance (BImp) sensors[14] have also been considered. Continuous non-invasive devices have been developed in recent decades to measure systolic blood pressure (SBP) continuously[15–18].

A novel measurement technique using radar sensor methodology, with continuous wave-doppler radar[19] and M-sequence ultra-wide band (UWB) sensor[20] techniques, is being developed to measure several vital signs, including BP. One group[21] uses an impulse-radio UWB radar to detect the heart-rate without requiring skin contact. However, this method is not able to measure BP without breath being held for 20 seconds during data acquisition. Continuous wave radar (CWR)-based techniques have also been used to detect heart sounds and to calculate heart rate[22]. A recent study used a CWR sensor to measure the carotid pulse at the left common carotid artery by placing a wearable antenna device at the neck[23]. Placing the sensor at the neck was uncomfortable for subjects during testing so the researchers evaluated CWR data acquired from sternal placement of the antenna[24] (measuring aortic arch activity), allowing the device to be hidden under clothing. BImp was measured across the shoulders[24].

One of the main goals of continuous ambulatory BP measurement is to investigate the effect of daily activities on BP. Many studies have been performed with the subjects in varied postures or undertaking exercise[25,26]. The PAT was measured as the

time interval between Electrocardiography (ECG) R-Peak and the steepest slope of the corresponding upstroke in the PPG signal obtained from a finger during cardiopulmonary exercise tests[27]. The SBPs were also measured simultaneously to find the relation between PAT and SBP using different regression methods[27]. . Another study tested dual-channel PPG signals from wrists and forearms to calculate the PTT and evaluated a method of removing motion artefacts from the SBP readings[28].

Accurate data analysis to derive useful information is as important as the sensor devices in BP calculation. Signal processing methods are needed and can be effective[29] in eliminating noise and motion artefacts. A discrete wavelet transform was employed to remove noise components of ECG signals[30]. Also, wavelet transform was used to monitor heart rate from non-contact CWR systems[31,32] and to detect the heart rate from contact-CWR signals[33].

In order to calculate the PTT, PAT and pre-ejection periods (PEP) need to be determined. As pre-load and after-load change in subjects, both PAT and PEP also vary but not always with the same magnitude or direction[34]. The PTT is calculated by subtracting the PEP from the PAT. The PAT is defined as the time interval between the ECG R-peak and the PPG peak. The PEP is the time between the opening of the aortic valve and the q-wave of the ECG[35]. Typically, the PEP is measured using pulsed doppler echocardiography (ECHO)[36]. Another technique is to measure the time between the B-point of the impedance cardiogram (ICG) (aortic valve opening) and the start of the Q-wave of the ECG[37]. The accuracy of the two techniques (particularly with respect to detecting aortic valve opening) has been compared using ECHO over the ascending aorta with subjects in either a supine position, or lying with their head up at 60 degrees[36], showing that ECHO calculated a non-significantly lower PEP than ICG. When the CWR antenna is placed on the sternum, the foot of the CWR signal, compared to the ICG B-point, is validated to correspond to the opening of the aortic valve[38–40].

Although ICG, BCG and PPG have been used as sensors to measure BP indirectly by peripheral or arterial readings, a pilot work shows that CWR can be a potential sensor to measure BP because the CWR sensor can be placed on the sternum to measure the opening of the aortic valve directly by targeting mechanical activity[19,23,24,38]. Peripheral arterial readings are affected by inaccuracies induced by vasomotion. This is particularly important for those patients who have long-term BP problems and require highly accurate measurements[19,23,24,38].Conveniently, these sensors can be worn under clothing. In the CWR sensors, the relation between PTT (extracting PEP from PAT) and BP has not been established. Further investigation is required to determine whether CWR, which can target central aortic pressure, is worth being pursued as a potential wearable technology.

In this study, we evaluate a method of calculating SBP whilst using data acquired from a combination of a CWR sensor on the sternum, a PPG sensor on the left earlobe, and ECG electrodes on the chest. The signals from these locations are less affected by body motion, organ artefact and vasomotor changes than from sensors placed in other locations. By placing the CWR antennae on the sternum, the CWR signal is hypothesized to acquire the arterial distension of the ventral side of the aortic arch, which matches the PEP obtained by the CWR antenna. A signal processing method, based on wavelet transform and an adaptive filter, are further developed for radar signals to detect accurate data, and regression methods are used to estimate SBP from PTTs derived from the proposed sensors signals.

Methods and Materials

Monitoring Setup

The block diagram in Figure 1a shows the setup used to record cardiac activities. It includes three main blocks to record CWR, PPG and ECG signals simultaneously. Figure 1a also shows the placements of two CWR antennae (the transmitter and receiver), on the aortic arch, two ECG electrodes on the chest and one PPG sensor on the left earlobe. The radar antennae's positions, R and T, represent the receiver and transmitter antennae.

Continuous Wave Radar

The CWR system, as shown in Figure 1a, includes two main transmitter and receiver parts. The transmitter, which is a local oscillator, emits a high-frequency wave signal $T_{cw}(t)$ through the body, , described by (1), using the transmitter antenna as an on-body sensor. The carrier frequency of the setup is around 900MHz.

The received signal $R_{cw}(t)$, defined by (2), includes the distance information d and periodic movement $m(t)$ of a target organ in its phase, i.e. aorta or heart. The information is derived using a mixer and a demodulator as two in-phase (I) and quadrature (Q) base-band components given by (3) and (4)[42]. The base-band signals are amplified and low pass filtered before being recorded.

$$T_{cw}(t) = E_T cos(\omega t + \phi) \tag{1}$$

$$R_{cw}(t) = E_R cos(\omega t - \omega_0(d + m(t)) + \theta) \tag{2}$$

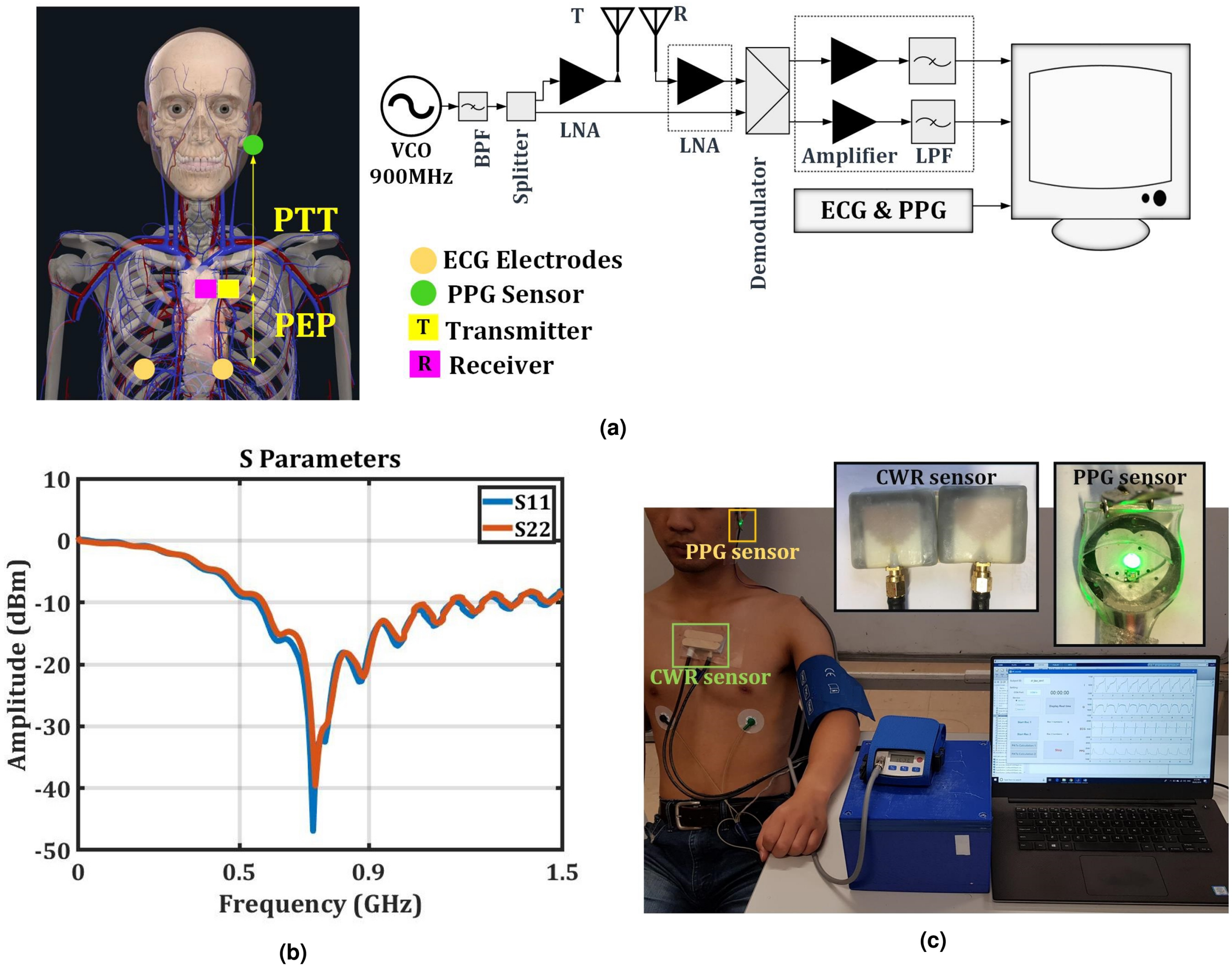

Figure 1. (a) The block diagram of the CWR setup and the placements of sensors on the body[41]. (b) S-parameters of the CWR antennae as on-body sensor. (c) The placement of CWR, ECG and PPG sensors on a participant seated on an exercise bike.

$$I(t) \approx sin(\omega_0 m(t) + \theta_0) \tag{3}$$

$$Q(t) \approx cos(\omega_0 m(t) + \theta_0) \tag{4}$$

In equations 1,2,3,4, ϕ is the phase noise in the setup, θ is a mixture of setup, body movement and environment phase noise, and θ_0 is an estimation of the combination of all phase noises.

Figure 1a shows the radar antennae placed at the sternum. Figure 1b shows the S parameter measurements for the antennae used. The S11 and S22 parameters are the ratio of the reflected power to the fed power at the transmitter and receiver antennae input which should be less than -10dB for proper antennae operation[24]. The S11 and S22 measurements show that they are below -15dB at 900MHz, which means the antennae works properly around the system's carrier frequency.

For any antenna used on or near the body, specified safety threshold values of the Specific Absorption Rate (1.6W/kg for 1g of tissue or 2.0W/kg averaged over 10g of tissue) must be obeyed[24]. The emitted power fed from the antenna to the body is around 2dBm (less than 2mW)[24].

Photoplethysmogram and Electrocardiogram

PPG signal is recorded to measure PAT parameters using a sensor which is placed on the left earlobe. The reason that the earlobe is selected to measure PPG is that the signal quality is good in this area of the body, i.e. clear signals can be measured and, that the recorded signal from the earlobe is steady with most body movements.

The ECG signal is measured as a reference signal using two electrodes which are placed on the chest. ECGs are used to derive heart beats and each heart beat's R-peak to measure time parameters, i.e. PAT and PEP.

All three different setups i.e. PPG, ECG and CWR are placed on the same device, and their outputs are recorded at the same time leading to synchronized data collection. Note that, to consider the hardware delay of each signal, a comparison is done with a reference device ECHO (i.e. ultra-sound M-mode wave) and the results show no considerable time shift in recorded waveforms.

The subjects wear a calibrated cuffed sphygmomanometer during tests to record their SBPs simultaneously. To synchronize the recorded signals with a BP measurement cuffed device, all data are labelled based on the same reference time information.

Signal Processing

The CWR, PPG and ECG signals are recorded simultaneously for all subjects. Figure 2a shows the block diagram of the proposed processing method to detect time parameters, i.e. PAT, PEP, and PTT.

For the first step, a Chebyshev type II band-pass filter (BPF) is used to remove the 50Hz components as well as noise and artefacts. To avoid the time shift caused by filtering, a zero-phase filtering algorithm is applied which helps to preserve the features of unfiltered signals exactly at the same time as the filtered wave-forms.

The pass-band of the BPF is chosen individually and automatically for each subject using the heart rate frequency (HRF) extracted from the individual subjects' PPG signals, and is fed into the analyses of the ECG, PPG and CWR. The varying band-pass filtering cut-off frequency method is required because this type of signal is prone to interference from respiration and tissue vasomotion, and individual subjects have different best cut-offs. To calculate the HRF, the floor of the first harmonic of Fast Fourier transform (FFT) is used. Then the BPF cut-off frequencies are defined for each signal as follows:

$$\begin{aligned} f_{pass_{low}EGC} = HRF, f_{pass_{high}ECG} = 30^{Hz}, \\ f_{pass_{low}CWR} = HRF, f_{pass_{high}CWR} = 2.5 \times HRF. \end{aligned} \tag{5}$$

After filtering, a combination of I and Q, i.e. arctangent of Q/I, is also calculated as described in (6).

$$\phi(t) = tan^{-1}Q(t)/I(t) = \omega_0 m(t) + \theta_0 \tag{6}$$

Wavelet

Sometimes using filtering alone may lead to a loss of some important timing information from a signal. In our study, utilizing BPFs may displace the foot of CWR signals, which leads to incorrect PEP measurements. Therefore, a wavelet transform is used to preserve considerable information and to remove the remained noise from the signals. The wavelet transform decomposes the signal into a number of scales having different frequency components, and analyses each scale with a certain resolution to obtain accurate features of the signal[33]. The sum of the overall time of the signal multiplied by a scaled and shifted version of the wavelet function, addressed as $H(a,b)$, is given as (7) and (8):

$$H(a,b) = \int_{-\infty}^{\infty} x(t)\Psi_{a,b}(t)dt \tag{7}$$

$$\Psi_{a,b}(t) = (1/\sqrt{a})\Psi^*((t-b)/a) \tag{8}$$

where, x(t) is the original signal, * denotes the complex conjugation, $\Psi_{a,b}(t)$ in the window function of the mother wavelet and $\Psi^*((t-b)/a)$ is its shifted and scaled version[30].

The selection of a particular wavelet function that closely matches the morphology of the signal under consideration is the most important factor for signal decomposition[30]. Since the CWR waveform is different from the known wavelet functions, a special mother wavelet function which matches our signals is designed and used as shown in Figure 2b. The blood pulse volume pumping from heart to the arterial tree leads to the aorta movements. The blood volume applies pressure to the walls of the aorta and depends on its elasticity and resistance, movements are generated. Hence, the movement pattern of the aorta is highly correlated (similar) to the aortic blood pressure (ABP)[43]. The aorta pressure waveform has two main peaks[43] which

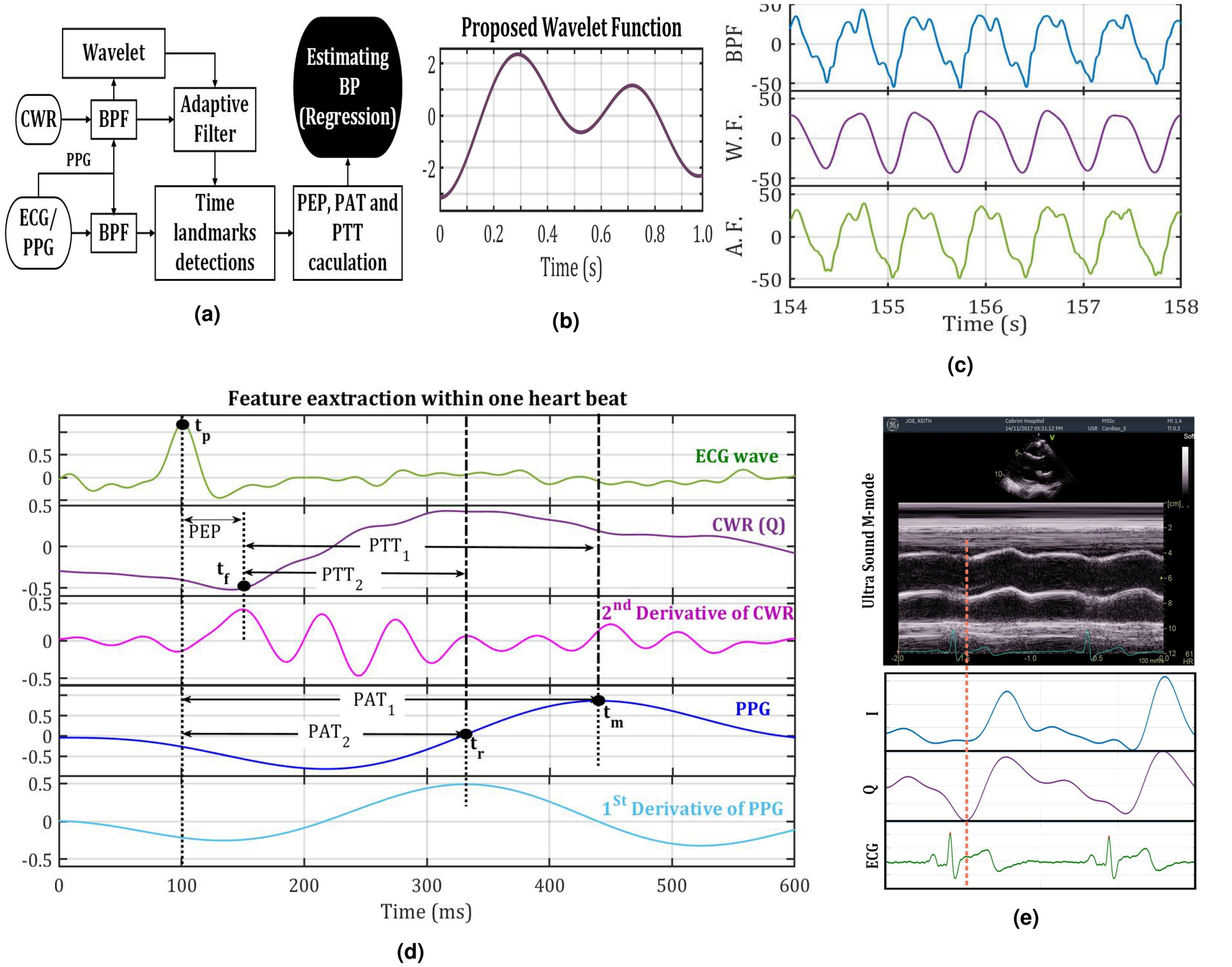

Figure 2. (a) The block diagram of used signal processing method to estimate BP from signals. (b) Proposed wavelet function design based on CWR signals waveform. (c) A sample of CWR signal (in-phase components) after BPF, wavelet transform and adaptive filtering for subject 1, standing position. (d) Samples of ECG, CWR, PPG, second derivative of CWRs, and first derivative of PPGs for subject 1 in standing position during the BP estimation. (e) A comparison between a sample of CWR signal (in-phase components) and M-mode signal of Ultra-sound.

can be used as the basic pattern to design the wavelet function. An algorithm is used to adapt a new wavelet to the discussed pattern by extracting the similar waveform on the interval of [0,1], which is also quite close to each beat of the CWR signal. The algorithm uses a least squares polynomial approximation of degree 6 to create the wavelet function[44]. Using the designed wavelet function, the CWR signals are decomposed down to level 12. Comparing the decomposed elements shows that the wavelet coefficients at scales 8 to 12 are closely representative of the heart rate for the general population; therefore, only the mentioned scales are used for reconstruction. It also leads to elimination of high frequency and very low-frequency noise sources, including respiration.

Adaptive Filter

By selecting the wavelet coefficients at special scales, unwanted noise is eliminated and smooths the signal, which is not negligible, in most cases. This selection may also lead to loss of some important time interval information; in other words, PEP measurements which are related to the foot of each beat of CWR signals. Therefore, adaptive filtering is used; the characteristics of which change in a way to obtain the best possible signal quality in spite of changing signal conditions[45]. An adaptive filter designs itself based on the characteristics of the input signal to the filter and a signal that represents the desired behavior of the filter on its input. Adaptive filters track the dynamic nature of a system and eliminate unwanted time-varying signals. Designing

the filter does not require any other frequency response information or specification. An adaptive algorithm is used to reduce the error between the output signal and the desired signal[46].

For CWR signal processing, the result of the wavelet transform is selected as the desired signal and the filtered signal is the input of the adaptive filter. In this case, those frequency components which are removed during wavelet transform remain; yet, since the wavelet-filtered signal is the desired signal, incorrect foot of signals can be removed. For the current study, an adaptive finite impulse response (FIR) filter uses a least mean squares (LMS) algorithm. The FIR filter weights are calculated using the LMS algorithm to minimize the mean square error (MSE) between the output signal and the desired signal[46]. The output of the adaptive filter is then used as a signal to derive PEP parameters. Figure 2c shows a sample of CWR signal after different levels of signal processing.

Blood Pressure Estimation

Figure 2d shows a sample of signals which are measured with the three sensors during the recording of heart activities. The PAT is selected as the time difference between the ECG R-peak (t_p) and the maximum of the PPG signal (t_m). It also can be assumed as the time difference between the ECG R-peak and the rising slope of the PPG signal (t_r).

As previously mentioned, a comparison between measured PEP from CWR and ICG sensors was undertaken to validate the precision of the PEPs[38–40]. In this study, the PEP measured from CWR signals after different levels of processing is compared with PEP measured simultaneously using ECHO i.e. ultra-sound M-mode wave to validate the accuracy and precision of the parameters, as shown in Figure 2e.TThis figure shows a high correlation between two measured PEPs and that the foot of the CWR signal is close to the foot of M-mode signal. In literature[37], it was shown that the QR interval can be assumed to be fixed; therefore, PEP is measured as the time interval between R-peak of ECG and the foot of CWR (t_f) signal.

Finally, the PTT—which is the difference between PAT and PEP—is measured to remove the effect of PEP from the PAT. After processing three ECG, PPG and CWR signals, the time parameters used for SBP estimation are calculated as following, and as highlighted in Figure 2d:

$$PAT_1 = t_p - t_m, \tag{9}$$

$$PAT_2 = t_p - t_r, \tag{10}$$

$$PEP = t_p - t_f, \tag{11}$$

$$PTT_1 = PAT_1 - PEP, \tag{12}$$

$$PTT_2 = PAT_2 - PEP. \tag{13}$$

Algorithm 1 R-peak detection

1: **procedure**
2: Inputs: ECG signals
3: Outputs: R-peaks
4: Initial R-peaks: the ECG signals' local maximums which have the $1/f_{pass_{low}.ECG}$ minimum distance from each other with 0.5 (a small value to observe nearly all peaks) minimum voltage drop from both sides (peak prominence). To calculate, we first find all local maximums using the first derivative of the ECG signal. Then eliminate the maximums with less than 0.5 peak prominence, and sort the maximums based on their amplitude (high to low). In order, measure the time interval between each maximum with the next maximum. If the time interval is less than the minimum distance ($1/f_{pass_{low}.ECG}$), remove the related peak. Store the remaining peaks as initial R-peaks.
5: mean values of R-peaks amplitudes: the mean value of the ECG signal at derived initial R-peaks in step 4.
6: Motion artefacts clearing: Remove those sections of the ECG signal which are more than the ECG's mean value + R-peaks amplitudes' mean values or less than the ECG's mean value - R-peaks amplitudes' mean values.
7: Conclusive R-peaks: the local maximums of the cleared ECG signal which have the $0.5/f_{pass_{low}.ECG}$ minimum distance from each other with $0.5 \times$ R-peaks amplitude mean values peak prominence. Repeat state 4.

To measure the time parameters, the location of the ECG R-peak is detected using Algorithm 1; then the boundaries of each heartbeat are measured.

To calculate PEP values, the Algorithm 2 is determined. As can be seen, a comparison is done between measured T_f elements and predefined thresholds $t_f min$, and $t_f max$ which are the reported ranges for PEP in[37].

Algorithm 2 PEP extraction

1: **procedure**
2: Inputs: I, Q, $tan^{-1}(I/Q)$, t_p, t_r, $t_f min$, $t_f max$ for n beats
3: Outputs: PEPs
4: Initial matrix T_f: find the local foots (time) of I, Q and $tan^{-1}(I/Q)$ signals during each heart beat using local maximum of second derivative method. Store them in $[T_f]_{3xn}$.
5: Thresholds: Exclude the elements of $[T_f]_{i,j}$ which cannot satisfy both the following conditions:

1. $t_p < [T_f]_{i,j} < t_r$,
2. $t_f min < [T_f]_{i,j} < t_f max$.

6: Calculate vector t_f: define t_f as the average of remained elements of T_f:

$$t_f = \underset{\mathbf{i}}{avg}\{[T_f]_{i,n}\} \quad (14)$$

7: Calculate PEPs: Calculate PEP values as the time interval between t_p and t_f ($PEP = t_p - t_f$).

To calculate PAT parameters, the rising slope and maximum of corresponding PPGs are measured as the maximum of the first derivative and the first local maximum of the signal, respectively.

The SBP values are estimated from PTTs using different regressions as follows:

$$SBP_{est} = a/PTT + b \quad (15)$$

$$SBP_{est} = a/PTT_1^2 + b/PTT_2^2 + c \quad (16)$$

where for equation 15, PTT can be PTT_1 or PTT_2. To determine the coefficients of the equations, cuff-based SBPs were measured and used as the calibration points of the models. Then the measured SBPs and the whole PATs and PTTs recorded from subjects were used to calculate the coefficients in a way that the estimated SBPs have the minimum difference from the measured SBPs. The coefficients a, b and c are calculated using minimum least square (MLS) fitting method. Different mathematical models have been suggested in literature to calculate BP from PTT or PAT[47,48]. Equation 15 is one of the most common with very high accuracy and simplicity as reported by researchers[47,48]. Most suggested models are based on using a one-time parameter. This led us to employ two-time variables in a model as in equation 16 to improve accuracy of the results.

Although the SBP estimation is done for each subject separately, in other words the equation coefficients are calculated separately, an algorithm is designed and used for regressions based on the two mentioned equations which do not need any calibration for coefficients or any tuning for an individual subject. We are using each three-minute cuffed sphygmomanometer measurements as calibration points to increase the calculation accuracy of a, b and c, so the errors will be reduced and the BP can be measured by equations. 15, and 16 continuously.

Experimental Protocol

Forty-three healthy volunteers participated in the experiments. Fifty two percent were male and 48% were female, between 40 and 65 years, (168+10) between 158 cm and 178 cm in height and weighed between 44 kg and 76kg with no participants reported of any previous cardiovascular problems. A written informed consent was required from each volunteer who participated in this study before starting the experiments. A video displaying testing procedures was recorded during one participant's test. Two written informed consents were obtained from the subject for study participation and for sharing any identifying image/video in an online open-access publication, respectively. Data collection was undertaken at Cabrini Hospital, Melbourne, Australia, (Oct. to Nov. 2017) and an in-room emergency physician ensured participants' safety during testing. All experiments were performed in accordance with the relevant guidelines and regulations and the required ethics permissions were obtained. (Cabrini Human Research Ethics Committee approval (07-19-06-17), ANZ clinical trial registration number ACTRN12617000774325.

Experimental conditions	PEP (ECG R-peak – CWR foot) Mean (SD) (ms)	PEP[37], included QR Mean (SD) (ms)
Sitting	64.80 (21.63)	108 (18)
Standing	66.22 (27.72)	117 (16)
Supine	47.24 (24.56)	95 (19)
Hand Grip	60.94 (30.67)	112 (17)
Light Cycling	55.56 (30.00)	115 (17)
Moderate Cycling	52.37 (21.27)	77 (20)
Heavy Cycling	47.96 (15.10)	66 (17)
Recovery 1	59.42 (18.40)	90 (25)
Recovery 2	53.80 (20.75)	NA

Table 1. The means and SDs for PEP measured for different experimental conditions.

ECG, PPG and CWR signals were measured simultaneously and the data from 43 participants were included in the analysis. The experiments were undertaken in two sessions. All volunteers participated in Session 1, while 26 subjects from among them were selected randomly to take part in Session 2. All subjects were asked to breathe normally during tests. The CWR data from five subjects were corrupted during signal recording because of the hardware failure, hardware data loss, loose cable connection or interrupted data collection; therefore, they were omitted for this work. Figure 1c shows the placement of CWR and PPG sensors for a participant seated on the exercise bike. The figure also shows the cuff placement and the software interface used for data recording.

Session 1 consisted of measuring all signals for three different postures; six minutes of sitting, six minutes of standing and six minutes in the supine position. The procedures for Session 2 were as follows:

- Holding handgrip for two minutes followed by a one-minute rest.
- Cycling with a fixed speed in three different bike resistant settings of light, moderate and heavy. Each cycle lasted for two minutes with a one-minute rest.
- Two recovery stages after the cycling tasks, during which subjects sat on the bike without any activity.

All ECG, PPG and CWR signals were recorded continuously during tests. Also, the subjects were asked to wear a calibrated cuffed sphygmomanometer during tests and their BP values were recorded every three minutes. The timing of cuff measurements was set so that the BP measurement happened once for each test and started after the second minute. The error of the hydrostatic effect was removed by keeping the cuff around the arm at the same horizontal height of the heart during all measurements[49].

Results

The SBP values of a healthy person cannot vary more than $\pm$10mmHg in two consecutive readings[50], while this variation may reach $\pm$15mmHg for anatomical abnormalities or measurement errors[50]. Since the probability of having anatomical abnormalities is very low, and the accuracy of the - cuffed sphygmomanometer used in this study is $\pm$3mmHg, we can consider measurements with a higher than 20mmHg(>15+3mmHg) deviation were caused by measurement errors. Therefore, all the cuff measurements with an error of more than 20 mmHg, and their related PTT or PAT values, were removed. The collected data were processed and analyzed in two independent categories: postures (sitting, standing and supine) and exercises (handgrip, light, moderate and heavy exercise, recovery 1 and recovery 2) categories independently. The values of $t_f min$ and $t_f max$ were chosen as 27ms and 78ms respectively[37].

Pre-ejection Period

Figures 3a and 3b show the box plots for two sessions of tests for all participants. Figure 3a shows the lowest PEP values, which were obtained in the supine posture. It can also be observed that the PEPs for sitting and standing positions are very close. A comparison of the results of the three different stages of cycling exercises, as shown in Figure 3b, shows a negative trend between the strength of activities and measured PEPs.

Table 1 represents the means and SDs values of the measured PEPs based on CWR signals for different conditions. Comparing the mean values of PEP for sitting and standing shows that there is a small rise (less than 1.42ms) for standing, while the PEP values drop considerably overall by 18.98ms to 47.24ms from standing to supine. From handgrip to heavy

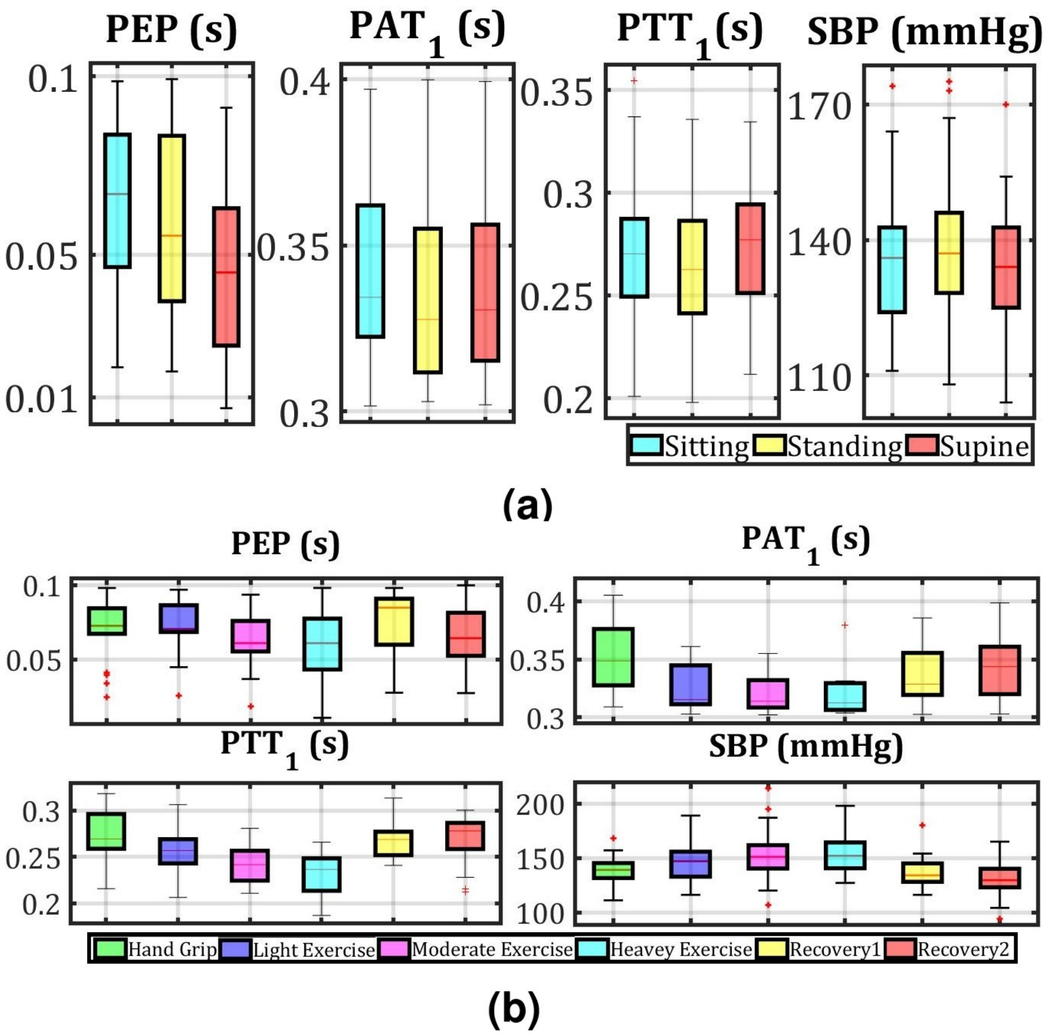

Figure 3. (a) Boxplots of PEP, PAT_1, PTT_1 and SBP median values for all participants, during three different posture tasks as sitting, standing and supine, and related measured SBPs using cuff. (b) Boxplots of PEP, PAT_1, PTT_1 and SBP median values for the participants, during six different exercise tasks as handgrip, light, medium and heavy exercise, recovery 1 and recovery 2.

cycling (increasing exercise stress) the PEPs fall from 60.94ms to 47.96ms, then return to normal values during recoveries (almost 55ms).

Considering the fact that the experiments' conditions are not exactly the same for different studies, it is not expected to observe the same PEP values. To validate the measured PEPs, a comparison was made between the trend of our results with the results of using ICG B-point based on reported work[37]. As can be seen the results show similar patterns for changing postures and exercise tasks.

Pulse Transit Time

Figures 3a and 3b show the box plots of PAT_1 and PTT_1 measurements for two sessions of tests for all participants and the boxplots of related BPs measured by the cuff. The box plots are used only to show the overall changes in extracted time parameters based on changing posture and exercise tasks. The measured PATs and PTTs in Figure 3a represent a slight difference for the different postures. Finding a clear trend between PAT and postures seems to be complicated. As can be seen, after calculating PTT (subtracting PEP from PAT) there is a similar trend for both PTT and SBP values due to changing the postures. The changes in PTTs for different postures show the noticeable effect of subtracting PEP from PATs to achieve a trustworthy pulse time delay parameter. Note that the relation between PTTs and SBPs should be considered in another approach, which is discussed in the next section.

	Algorithm	Parameter	CEP_7[1]	CEP_10[2]	CEP_15[3]	CEP_1015[4]	CEP_1520[5]	CEP_20[6]
Posture	Equation 15	PAT1	76.02	79.27	84.96	5.69	2.03	0.41
		PAT2	65.74	70.83	75.00	4.17	2.31	0
		PTT1	81.30	84.55	92.28	7.72	2.44	0.41
		PTT2	73.08	78.63	85.47	6.84	1.71	0
Exercise	Equation 15	PAT1	61.48	66.67	76.30	9.63	3.70	5.93
		PAT2	56.30	59.26	71.11	11.85	4.44	4.44
		PTT1	71.20	75.20	80.80	5.60	3.20	4.80
		PTT2	55.80	67.39	78.99	11.59	3.62	6.52
	Equation 16	PAT1 & PAT2	61.48	63.70	71.11	7.41	4.44	4.44
		PTT1 & PTT2	76.09	76.81	82.61	5.80	1.45	5.07

[1] CEP<7mmHg
[2] CEP<10mmHg
[3] CEP<15mmHg
[4] 10mmHg<CEP<15mmHg
[5] 15mmHg<CEP<20mmHg
[6] 20mmHg<CEP

Table 2. Accuracy percentage based on PAT_1 and PAT_2, using Cumulative error percentage (CEP).

Figures 3a and 3b show both PAT and PTT have negative trends with respect to exercise stress during exercise tasks, while BPs have the same trend. While PATs and PTTs decrease when the bike exercise was intense, their related BPs increase. The changes for both PAT and PTT were quite clear, which means that the effect of exercise is sufficiently significant despite the influence of PEPs.

Blood Pressure Calculation

Figures 4a and 4b show the measured SBPs based on their related PTT and PAT values during three different posture tasks. As seen, there is not a clear relation between extracted PAT values with SBPs, while PTTs and SBPs remain negatively correlated despite having same trend due to posture changing.

Similarly, figures 4c, 4d, 4e and 4f show the cuff-based measured SBPs against PAT_1 and PTT_1 for exercise tasks. Both PATs and PTTs illustrate an inverse relation to SBPs during exercise, which was also observed for their trends in the previous section. In Figures 4e and 4f the estimated inverse relation between SBPs and PATs/PTTs using equation 15 are presented as curves. The curves are plotted (based on equation 15) for each subject independently, using the MATLAB curve-fitting tool. As can be seen, the fitted curves for different subjects based on PATs are more scattered than for those based on PTTs. The calculated curves for SBP-PTTs are more linear and coherence than SBP-PATs. This leads to a noticeable improvement in calculating SBPs based on PTTs due to eliminating the PEP effect from PATs.

Figures 5a and 5b show the Bland-Altman (BA) plots with limits of agreement (LOA) for all estimated SBPs using equations 15, based on cuff-based measured SBPs, and time parameters including PAT_1 and PAT_2, PTT_1, and PTT_2, for different posture and exercise tasks. As can be seen, there is a considerable improvement in calculating SBPs using PTTs compared to that of using the PATs.

In Figure 5c the results of SBP estimation with equation 16, using the combination of PAT_1 and PAT_2/PTT_1 and PTT_2, are shown. Equation 16 cannot be applied for postures because the number of points are not enough (less than four for each participant) and this leads to over-fitting. The combination of PTTs compared with PATs provides significant improvement in terms of SBPs calculation accuracy.

Table 2 presents the accuracy percentage for estimated SBPs using PAT_1, PAT_2, PTT_1, PTT_2, and cuff SBPs, which are based on equations 15 and 16 for different postures and exercise tasks. The accuracy is calculated using the cumulative error percentage (CEP) of less than 7mmHg, less than 10 mmHg, less than 15mmHg, between 10mmHg and 15mmHg, between 15mmHg and 20mmHg, and more than 20mmHg. The CEP less than 7mmHg is considered based on the newly established IEEE standard on continuous blood pressure measurement system[2]. By comparing the results of estimated SBP based on PATs and PTTs, it can be seen that eliminating PEP leads to an almost 9% improvement. Another observation is that the accuracy percentages for the posture session are higher than for the exercise tasks. This is because recorded signals during posture measurements are less noisy, due to the participants' steadiness, compared with exercise tasks where subjects' body movements increase due to cycling.

Table 2 illustrate that the results of using equation 15 based on the first order of PTT_1 are slightly better than using PTT_2 for the same equation or using the second order equation 16 based on PTT_1 and PTT_2.

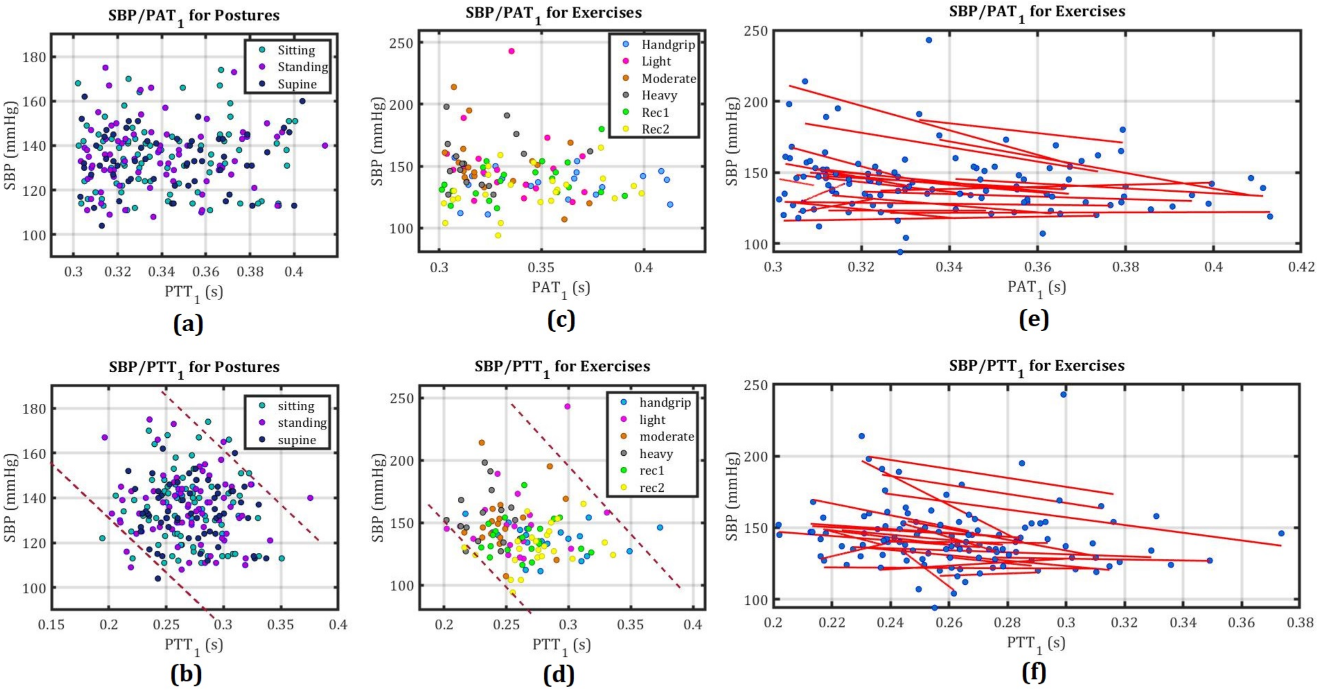

Figure 4. (a) $SBPs/PAT_1$ for three posture tasks, (b) $SBPs/PTT_1$ for three posture tasks. (c),(d) $SBPs/PAT_1$ for six exercise tasks, (e),(f) $SBPs$ and PTT_1 for six exercise tasks, and individually fitted curves for each subject using equation 15.

Considering the complexity of the calculation for equation 16, using two variables from the same signals i.e. PTT_1 and PTT_2 does not necessarily leading to the optimum solution.

Discussion and Conclusion

The proposed method in this study investigates continuous BP estimation based on PTT measurements for the first time using on-body radar sensors on the chest and PPG signals from the ear. By positioning the CWR sensor on the sternum and evaluating its signal, PEP is measured as the difference between the foot of CWR signal and ECG R-peak. Comparisons between the effects of changing posture and different exercise tasks on PTT, PAT and their related SBP were conducted. The results show that the removal of PEP values from PATs of PPG signals to estimate the SBP based on PTT, leads to more accuracy. Two different regression methods (first order and second order equations using one variable and two variables respectively), based on equations 15 and 16, are implied to calculate SBPs from PTTs and PATs.

The proposed CWR sensors have the ability to observe mechanical movements of the aortic valve (the aortic arch opening) similar to ECHO, which is considered as a reference method to observe aortic activities with the highest accuracy in PEP extraction[36]. Despite the focus on ICG technologies, CWR presents a low-power, continuous and potentially wearable system with minimal body contact to monitor aortic valve mechanical activities directly. In addition, compared to other pulse wave monitoring methods, such as PPG, CWR can provide central elastic aorta monitoring, removing the peripheral vasomotion-induced inaccuracies, which requires higher quality in the design of CWR sensors for recordings and processing in future work. This shows the potential of the CWR-based system to provide a simple wearable device, which can monitor both PEPs and PTTs (in results, beat-to-beat SBPs).

Data are collected during different posture and exercise tasks considering their effects on time parameters and BPs separately. This leads to better understanding of changing posture without having activity impacts and vice versa. Removing the effect of PEPs from PATs leads to a clear negative relation between PTTs and SBPs for various postures, and a more coherent negative relation (as proved in other research studies[7,27]) for exercise tasks. It should be considered that the variations of time intervals for different postures significantly relate to each subject's specifications. In addition, the BP variations are very low for different postures[12,26]. The experimental conditions are designed to observe higher than normal BP values, while there is a need to study the decreasing trends of BP as well. In addition, investigating continuous data (beat-to-beat) can provide a better understanding of the system and its time parameters.

The feet of CWR signals using the maximum of the second derivative, compared with[37] presents the PEP values[37]. The output of CWR contains three main signals as I, Q and arctangent Q/I. Instead of using these CWR signals separately, the

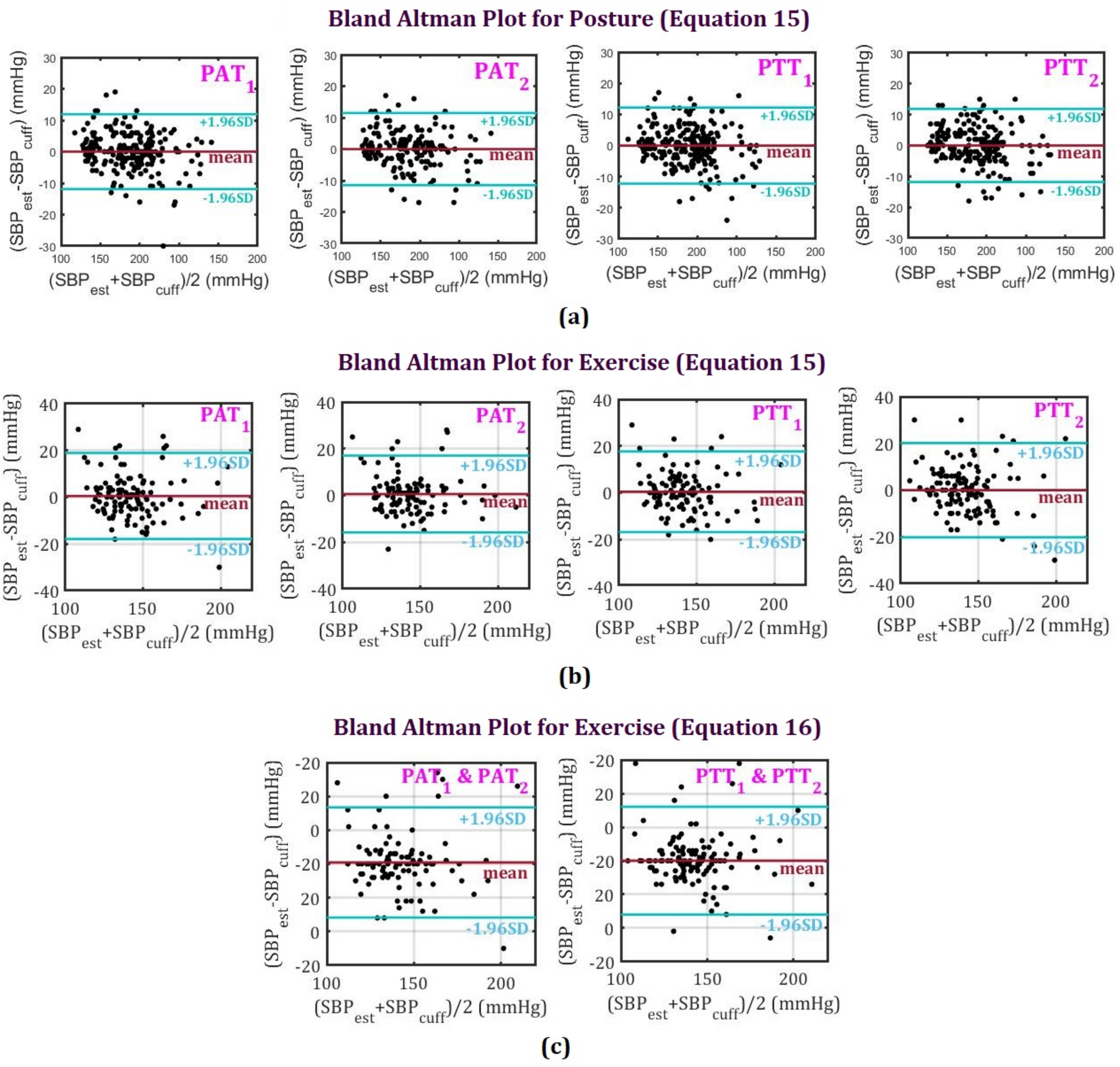

Figure 5. Bland Altman (BA) with limits of agreement (LOA) (mean $\pm$ 1.96*standard deviation (SD)) plots using estimated *SBP* and cuff-based measured *SBP* based on PAT_1, PAT_2, PTT_1 and PTT_2. (a) BA plots for posture tasks using equation 15. (b) BA plots for exercise tasks using equation 15. (c) BA plots for exercise tasks using equation 16.

combination of I, Q and arctangent-based extracted PEPs is presented to obtain more accurate values. To derive PTT parameters, the PEPs are subtracted from PATs. The PATs are measured as the rising slope and maximum of corresponding PPGs by calculating the maximum of the first derivative and the first local maximum of the PPG signal. The proposed signal processing methods based on wavelet and adaptive filtering techniques are employed to clear radar signals by removing unwanted noise and artefacts without causing any time shift. A BPF is designed to omit unwanted frequencies considering each subject's heart rate which allows more accurate respiration/movement artefact's filtering.

In some cases, the radar signals are not as reproducible in shape, especially during exercise tasks. There may be some other factors such as the heart size, position within the thorax and orientation, and respiration which may affect the signal and the place of its foot, subject by subject[24]. One other challenge to achieve a clear radar signal is the shape of antennae as an on-body sensor, and also the way that they are attached to the body. Redesigning the current antennae to be small and flexible, targeting

more reliable signal detection along with applying more advanced signal processing methods to extract PEP values with higher accuracy, will be investigated in future works.

The SBP calculations based on PATs and PTTs are investigated separately using two different mathematical models. The results show a noticeable effect of PEP-correction in the SBP calculation accuracy. For both experimental conditions, an overall improvement of 10% is achieved using PTTs compared to PATs. The highest improvement is obtained using equation 15 based on PTT_1s which also has the advantage of simplicity. Based on the results represented in this study, the potential superiority of CWR-based PEP extraction for different purposes such as SBP calculations is demonstrated.

Sometimes the PEP can change in an opposite direction to the PAT during BP changes due to the variations in preload and afterload[34]. Note that the PEP (in parallel PTT) can be influenced, not only by position or activity of the subject, but also by other parameters such as heart rate, vessel elasticity, age, body fat and gender. Adding the effect of these parameters to the mathematical regression methods could provide more accurate SBPs estimation, which will be studied in future works.

References

1. Perkovic, V., Huxley, R., Wu, Y., Prabhakaran, D. & MacMahon, S. The burden of blood pressure-related disease: a neglected priority for global health. *Hypertension* **50**, 991–997 (2007).
2. Ieee standard for wearable cuffless blood pressure measuring devices. *IEEE Std 1708-2014* 1–38, DOI: 10.1109/IEEESTD.2014.6882122 (2014).
3. Beevers, G., Lip, G. Y. & O'brien, E. Blood pressure measurement: Part i—sphygmomanometry: factors common to all techniques. *Bmj* **322**, 981–985 (2001).
4. Ogedegbe, G. & Pickering, T. Principles and techniques of blood pressure measurement. *Cardiol. clinics* **28**, 571–586 (2010).
5. Hodgkinson, J. *et al.* Relative effectiveness of clinic and home blood pressure monitoring compared with ambulatory blood pressure monitoring in diagnosis of hypertension: systematic review. *BMJ* **342**, DOI: 10.1136/bmj.d3621 (2011). https://www.bmj.com/content/342/bmj.d3621.full.pdf.
6. Scheer, B. V., Perel, A. & Pfeiffer, U. J. Clinical review: complications and risk factors of peripheral arterial catheters used for haemodynamic monitoring in anaesthesia and intensive care medicine. *Critical Care* **6**, 199 (2002).
7. Wong, M. Y.-M., Poon, C. C.-Y. & Zhang, Y.-T. An evaluation of the cuffless blood pressure estimation based on pulse transit time technique: a half year study on normotensive subjects. *Cardiovasc. engineering* **9 1**, 32–8 (2009).
8. Martin, S. L.-O. *et al.* Weighing scale-based pulse transit time is a superior marker of blood pressure than conventional pulse arrival time. *Sci. reports* **6**, 39273 (2016).
9. Anchan, R. *Estimating pulse wave velocity using mobile phone sensors*. Bachelor's thesis, Edith Cowan University (2011).
10. Hsu, Y.-P. & Young, D. J. Skin-coupled personal wearable ambulatory pulse wave velocity monitoring system using microelectromechanical sensors. *IEEE Sensors J.* **14**, 3490–3497 (2014).
11. Lee, Y.-J. *et al.* Magneto-plethysmographic sensor for peripheral blood flow velocity. *IEEE Sensors journal* **14**, 1341–1342 (2014).
12. Thomas, S. S. *et al.* Biowatch: A noninvasive wrist-based blood pressure monitor that incorporates training techniques for posture and subject variability. *IEEE journal biomedical health informatics* **20**, 1291–1300 (2016).
13. Holz, C. & Wang, E. J. Glabella: Continuously sensing blood pressure behavior using an unobtrusive wearable device. *Proc. ACM on Interactive, Mobile, Wearable Ubiquitous Technol.* **1**, 58 (2017).
14. F. Heydari, T. W. K. W. K. J. J. M. R., M. P. Ebrahim & Yuce, M. R. Continuous cuffless blood pressure measurement using body sensors. In *13th International Conference of Body Area Network (BodyNets) of EAI, Finland, Oulu* (2018).
15. Chen, W., Kobayashi, T., Ichikawa, S., Takeuchi, Y. & Togawa, T. Continuous estimation of systolic blood pressure using the pulse arrival time and intermittent calibration. *Med. Biol. Eng. Comput.* **38**, 569–574, DOI: 10.1007/BF02345755 (2000).
16. Proença, J. D. L. *Improved disease management of heart failure patients using the pulse wave velocity methodology*. Ph.D. thesis, Coimbra (2010).
17. Surendhra, G. & Jayanthi, T. Cuff less continuous non-invasive blood pressure measurement using pulse transit time measurement. *Recent Dev. Eng. Technol.* **2**, 86–91, DOI: http://www.ijrdet.com/Volume2Issue1.php (2014).
18. Mukkamala, R. *et al.* Toward ubiquitous blood pressure monitoring via pulse transit time: Theory and practice. *IEEE Transactions on Biomed. Eng.* **62**, 1879–1901, DOI: 10.1109/TBME.2015.2441951 (2015).

19. Muehlsteff, J., Thijs, J., Pinter, R., Morren, G. & Muesch, G. A handheld device for simultaneous detection of electrical and mechanical cardio-vascular activities with synchronized ecg and cw-doppler radar. In *Engineering in Medicine and Biology Society, 2007. EMBS 2007. 29th Annual International Conference of the IEEE*, 5758–5761 (IEEE, 2007).

20. Helbig, M., Zender, J., Ley, S. & Sachs, J. Simultaneous electrical and mechanical heart activity registration by means of synchronized ecg and m-sequence uwb sensor. In *Antennas and Propagation (EuCAP), 2016 10th European Conference on*, 1–3 (IEEE, 2016).

21. Lee, Y. *et al.* A novel non-contact heart rate monitor using impulse-radio ultra-wideband (ir-uwb) radar technology. *Sci. reports* **8**, 13053 (2018).

22. Will, C. *et al.* Radar-based heart sound detection. *Sci. reports* **8**, 11551 (2018).

23. Buxi, D., Redouté, J.-M. & Yuce, M. R. Cuffless blood pressure estimation from the carotid pulse arrival time using continuous wave radar. In *Engineering in Medicine and Biology Society (EMBC), 2015 37th Annual International Conference of the IEEE*, 5704–5707 (IEEE, 2015).

24. Buxi, D., Redouté, J.-M. & Yuce, M. R. Blood pressure estimation using pulse transit time from bioimpedance and continuous wave radar. *IEEE Transactions on Biomed. Eng.* **64**, 917–927 (2017).

25. Zheng, Y., Poon, C. C., Yan, B. P. & Lau, J. Y. Pulse arrival time based cuff-less and 24-h wearable blood pressure monitoring and its diagnostic value in hypertension. *J. medical systems* **40**, 195 (2016).

26. Muehlsteff, J., Aubert, X. & Morren, G. Continuous cuff-less blood pressure monitoring based on the pulse arrival time approach: The impact of posture. In *Engineering in Medicine and Biology Society, 2008. EMBS 2008. 30th Annual International Conference of the IEEE*, 1691–1694 (IEEE, 2008).

27. Wibmer, T. *et al.* Pulse transit time and blood pressure during cardiopulmonary exercise tests. *Physiol. research* **63** (2014).

28. Wang, Y., Liu, Z. & Ma, S. Cuff-less blood pressure measurement from dual-channel photoplethysmographic signals via peripheral pulse transit time with singular spectrum analysis. *Physiol. measurement* **39**, 025010 (2018).

29. Ghasemzadeh, H., Ostadabbas, S., Guenterberg, E. & Pantelopoulos, A. Wireless medical-embedded systems: A review of signal-processing techniques for classification. *IEEE Sensors J.* **13**, 423–437 (2013).

30. Sahoo, S., Biswal, P., Das, T. & Sabut, S. De-noising of ecg signal and qrs detection using hilbert transform and adaptive thresholding. *Procedia Technol.* **25**, 68–75 (2016).

31. Mercuri, M. *et al.* A direct phase-tracking doppler radar using wavelet independent component analysis for non-contact respiratory and heart rate monitoring. *IEEE transactions on biomedical circuits systems* **12**, 632–643 (2018).

32. Li, M. & Lin, J. Wavelet-transform-based data-length-variation technique for fast heart rate detection using 5.8-ghz cw doppler radar. *IEEE Transactions on Microw. Theory Tech.* **66**, 568–576 (2018).

33. Ebrahim, M. P., Heydari, F., Redoute, J.-M. & Yuce, M. R. Accurate heart rate detection from on-body continuous wave radar sensors using wavelet transform. In *2018 IEEE SENSORS*, 1–4 (IEEE, 2018).

34. Zhang, G., Gao, M., Xu, D., Olivier, N. B. & Mukkamala, R. Pulse arrival time is not an adequate surrogate for pulse transit time as a marker of blood pressure. *J. applied physiology* **111**, 1681–1686 (2011).

35. Ebrahim, M. P., Heydari, F., Redoute, J.-M. & Yuce, M. R. Pre-ejection period (pep) estimation based on r–wave in ecg and on-body continuous wave radar signal during daily activities. In *13th International Conference of Body Area Network (BodyNets) of EAI, Finland, Oulu* (2018).

36. Cybulski, G., Michalak, E., Koźluk, E., Piątkowska, A. & Niewiadomski, W. Stroke volume and systolic time intervals: beat-to-beat comparison between echocardiography and ambulatory impedance cardiography in supine and tilted positions. *Med. Biol. Eng. Comput.* **42**, 707–711 (2004).

37. van Lien, R., Schutte, N. M., Meijer, J. H. & de Geus, E. J. Estimated preejection period (pep) based on the detection of the r-wave and dz/dt-min peaks does not adequately reflect the actual pep across a wide range of laboratory and ambulatory conditions. *Int. J. Psychophysiol.* **87**, 60–69 (2013).

38. Buxi, D., Dugar, R., Redouté, J.-M. & Yuce, M. R. Comparison of the impedance cardiogram with continuous wave radar using body-contact antennas. In *Engineering in Medicine and Biology Society (EMBC), 2017 39th Annual International Conference of the IEEE*, 693–696 (IEEE, 2017).

39. Buxi, D. *et al.* Systolic time interval estimation using continuous wave radar with on-body antennas. *IEEE J. Biomed. Heal. Informat.* **22**, 129–139 (2018).

40. Buxi, D., Redouté, J.-M. & Yuce, M. R. Systolic time interval estimation at the sternum using continuous wave radar with body-contact antennas. In *Wearable and Implantable Body Sensor Networks (BSN), 2017 IEEE 14th International Conference on*, 87–90 (IEEE, 2017).

41. 3d4medical ltd, complete anatomy (version 4.2.2(5265)) (2019).

42. Pour Ebrahim, M., Sarvi, M. & Yuce, M. R. A doppler radar system for sensing physiological parameters in walking and standing positions. *Sensors* **17**, 485 (2017).

43. Esper, S. A. & Pinsky, M. R. Arterial waveform analysis. *Best Pract. & Res. Clin. Anaesthesiol.* **28**, 363–380 (2014).

44. Misiti, M., Misiti, Y., Oppenheim, G. & Poggi, J.-M. *Wavelets and their Applications* (John Wiley & Sons, 2013).

45. Real-time dsp, chapter 8, adaptive filters. http://www.eas.uccs.edu/~mwickert/ece5655/.

46. Overview of adaptive filters and applications. https://au.mathworks.com/help/dsp/ug/overview-of-adaptive-filters-and-applications.html.

47. Sharma, M. *et al.* Cuff-less and continuous blood pressure monitoring: a methodological review. *Technologies* **5**, 21 (2017).

48. Zhang, Q., Zhou, D. & Zeng, X. Highly wearable cuff-less blood pressure and heart rate monitoring with single-arm electrocardiogram and photoplethysmogram signals. *Biomed. engineering online* **16**, 23 (2017).

49. Goldsmith, J. Hemodynamics and cardiology. *Congenit. Hear. Dis.* **4**, 128–129 (2009).

50. Clark, V. L. & Kruse, J. A. Clinical methods: the history, physical, and laboratory examinations. *Jama* **264**, 2808–2809 (1990).

Acknowledgements

The authors would like to thank previous members of the Biomedical Integrated Circuits and Sensors (BICS) Laboratory, Monash University, for their help in developing the prototype and the antenna for data collections. The authors would like to thank all participants for their collaboration as human subjects. The authors gratefully acknowledge the support of the Monash Institute of Medical Engineering for this project.

Author contributions statement

M.P.E. developed the algorithm to analyze the results. M.R.Y. and J.M.R. initiated the project. M.P.E., M.R.Y., J.M.R., F.H and T.W. built the experimental setup. M.P. and F.H. conducted the trial. K.J. lead the trial. K.W. organized ethics for data collection. K.J. and K.W. helped in validating data. All authors reviewed the manuscript.

Additional information

Competing interests:

The authors have no competing interests.

Funding:

This work is supported in part by an ARC Linkage Project (LP160101823). M. R. Yuce's work is supported by Australian Research Council Future Fellowships Grant FT130100430.
